# Conserved and Distinct Elements of Phagocytosis in Human and *C. elegans*

**DOI:** 10.3390/ijms22168934

**Published:** 2021-08-19

**Authors:** Szilvia Lukácsi, Zsolt Farkas, Éva Saskői, Zsuzsa Bajtay, Krisztina Takács-Vellai

**Affiliations:** 1 MTA-ELTE Immunology Research Group, Eötvös Loránd Research Network (ELKH), Eötvös Loránd University, Pázmány Péter s. 1/C, 1117 Budapest, Hungary; lukacsisz@student.elte.hu (S.L.); bajtay.zsuzsanna@ttk.elte.hu (Z.B.); 2 Department of Biological Anthropology, Eötvös Loránd University, Pázmány Péter s. 1/C, 1117 Budapest, Hungary; zsolt.farkas@ttk.elte.hu (Z.F.); eva.saskoi@ttk.elte.hu (É.S.); 3 Department of Immunology, Eötvös Loránd University, Pázmány Péter s. 1/C, 1117 Budapest, Hungary

**Keywords:** endocytosis, phagocytosis, efferocytosis, apoptosis, *Caenorhabditis elegans*, pattern recognition receptors, innate immunity

## Abstract

Endocytosis provides the cellular nutrition and homeostasis of organisms, but pathogens often take advantage of this entry point to infect host cells. This is counteracted by phagocytosis that plays a key role in the protection against invading microbes both during the initial engulfment of pathogens and in the clearance of infected cells. Phagocytic cells balance two vital functions: preventing the accumulation of cell corpses to avoid pathological inflammation and autoimmunity, whilst maintaining host defence. In this review, we compare elements of phagocytosis in mammals and the nematode *Caenorhabditis elegans*. Initial recognition of infection requires different mechanisms. In mammals, pattern recognition receptors bind pathogens directly, whereas activation of the innate immune response in the nematode rather relies on the detection of cellular damage. In contrast, molecules involved in efferocytosis—the engulfment and elimination of dying cells and cell debris—are highly conserved between the two species. Therefore, *C. elegans* is a powerful model to research mechanisms of the phagocytic machinery. Finally, we show that both mammalian and worm studies help to understand how the two phagocytic functions are interconnected: emerging data suggest the activation of innate immunity as a consequence of defective apoptotic cell clearance.

## 1. Introduction

Phagocytosis and phagocytes were discovered by the Russian biologist Elia Metchnikoff in the late 19th century, and for this discovery, he was awarded the Nobel Prize in physiology and medicine in 1908, together with Paul Ehrlich. Phagocytic clearance plays a central role during normal tissue turnover, remodelling of embryonic tissues, the development of the immune system, and the resolution of inflammation [1].

Innate immunity is the evolutionary ancient part of the immune system that ensures immediate protection against invading microbes. Whereas the adaptive immune system emerged only during early vertebrate evolution, elements of innate immunity provide the protection of multicellular organisms in most invertebrate animals, including worms. These components might include physical barriers, secreted antimicrobial molecules or cells with a phagocytic capacity [2]. It is important to note, however, that even in vertebrates, adaptive immunity is strongly dependent on the activity of innate immune cells, and the co-evolution of the two arms formed the diverse and efficient immune system present in mammals.

The free-living nematode *C. elegans* coexists with a variety of pathogens such as bacteria, fungi and viruses in the soil. Pathogens can attack and infect the nematode via the cuticle and epidermis, the uterus, the rectum, or they might colonize the intestine. As *C. elegans* does not possess specialized immune cells, the above tissues also have a function in host defence. Additionally, the cuticle and the pharyngeal grinder often serve as mechanical barriers ensuring the first line of protection against microbes [3].

In humans the innate immune system consists of various cells, among them are neutrophils, monocytes, macrophages and immature dendritic cells known as professional phagocytes. These cells have a dual role in immunity: they are both eliminating the threat of non-self molecules and pathogens, and process self-antigens derived from engulfed apoptotic cells [4].

Tissue-resident phagocytes, e.g., macrophages in a non-inflamed environment, recognize, internalize and eliminate the cellular debris and apoptotic cells that are generated by normal development and tissue repair without inducing an inflammatory immune response. Neutrophil granulocytes are recruited in a large number to the sites of infection, where they phagocytose the pathogens, then undergo apoptosis. Apoptotic neutrophils are ingested by macrophages preventing the release of toxic molecules into the environment. Apoptotic cell recognition is mediated through the detection of molecules that are normally absent on live and healthy cells. The phagocytosis of apoptotic neutrophils induces anti-inflammatory cytokine synthesis by macrophages [5].

In contrast to this, the phagocytosis of microbes triggers a pro-inflammatory immune response. Microbial molecules including bacterial and fungal wall constituents, viral capsid components or pathogen-derived nucleotides are recognized by pattern recognition receptors (PRRs). The ligation of PRRs triggers pro-inflammatory and antimicrobial responses by inducing the activation of transcription factors, such as NF-κB, or interferon regulatory factors which leads to the elimination of the invading organism [6].

Activated phagocytes produce large amounts of reactive species to eliminate pathogens. The two main families of these molecules are reactive oxygen (ROI) and nitrogen intermediates (RNI). ROI are generated by the activation of the NADPH oxidase (NOX) during the so-called oxidative burst. The superoxide radical (O_2_^−^) is formed as a product of the enzymatic activities of NOX and O_2_^−^ is transformed by superoxide dismutase (SOD) enzymes to hydrogen peroxide (H_2_O_2_), which can be further converted to the hydroxyl radical (OH^−^). RNI molecules are produced during the conversion of L-arginine by the NO synthase (NOS) enzyme [7].

Phagocytes actively maintain an alkaline pH in their phagosomes via the activity of NOX, to kill microbial pathogens. The genetically determined lack of NOX in phagocytes leads to the development of chronic infections in various organs [8]. The redox homeostasis is maintained by antioxidant enzymes, including superoxide dismutase, catalase as well as non-enzymatic antioxidant molecules [9].

The insufficient regulation of reactive oxygen species leads to a state of oxidative stress. Harmful effects of ROI and RNI species are present both on the molecular (protein and lipid oxidation, DNA damage) and cellular level (effect on signal transduction, cell membrane functions and gene expression) [10,11]. Oxidative stress plays a crucial role in the development and maintenance of chronic inflammation and thus contributes to pathological conditions such as cardiovascular diseases, diabetes, neurodegenerative diseases or cancer [12]. Reactive oxidants affect all stages of the inflammatory response, including the release of molecules acting as endogenous danger signals, their sensing by innate immune receptors like TLRs and NLRs and the activation of signalling pathways initiating the immune response [13].

Phagocytosis provides crucial functions in immune defence: it is essential to clear pathogens and cellular debris in eukaryotic organisms [14]. The latter process, clearance of dying, apoptotic cells is essential in mammals to avoid pathological inflammation. The nematode *Caenorhabditis elegans* has been known as a powerful model to study cell death processes [15,16]. As cell death and cell clearance pathways are highly conserved between nematodes and mammals, the nematode has also emerged as a model organism to study the cause of diseases related to the misregulation of these processes [17]. In this review we focus on components and mechanisms of phagocytosis in mammals and in *C. elegans*. We show that genetic pathways involved in phagocytosis of cell corpses are highly conserved and studies on the worm contributed significantly to our understanding of apoptotic clearance mechanisms in humans. Our aim is also to compare how pathogen recognition and uptake is managed in the two systems: while in mammals pathogens are directly recognized and bound by a range of receptors of innate immune cells, in the worm, in the absence of direct pathogen recognition, the innate immune response is activated by cellular damage as a consequence of pathogen infection.

Interestingly, worms can avoid pathogen attacks by sensing metabolites of microbes via multiple sensory neurons, showing that innate immunity is regulated by the nervous system. Thus, the tiny worm can also be used as a model to study neuroimmunology, which is a dynamically developing field focusing on the connection of the nervous and immune systems. Finally, we summarize emerging data analyzing poorly understood interconnections between the two phagocytic functions, apoptotic clearance and initiation of the innate immune response against pathogens. Recent data obtained in both worms and mammals suggest activation of innate immunity as a consequence of defective apoptotic clearance [18].

## 2. Forms of Endocytosis

**Figure 1:**
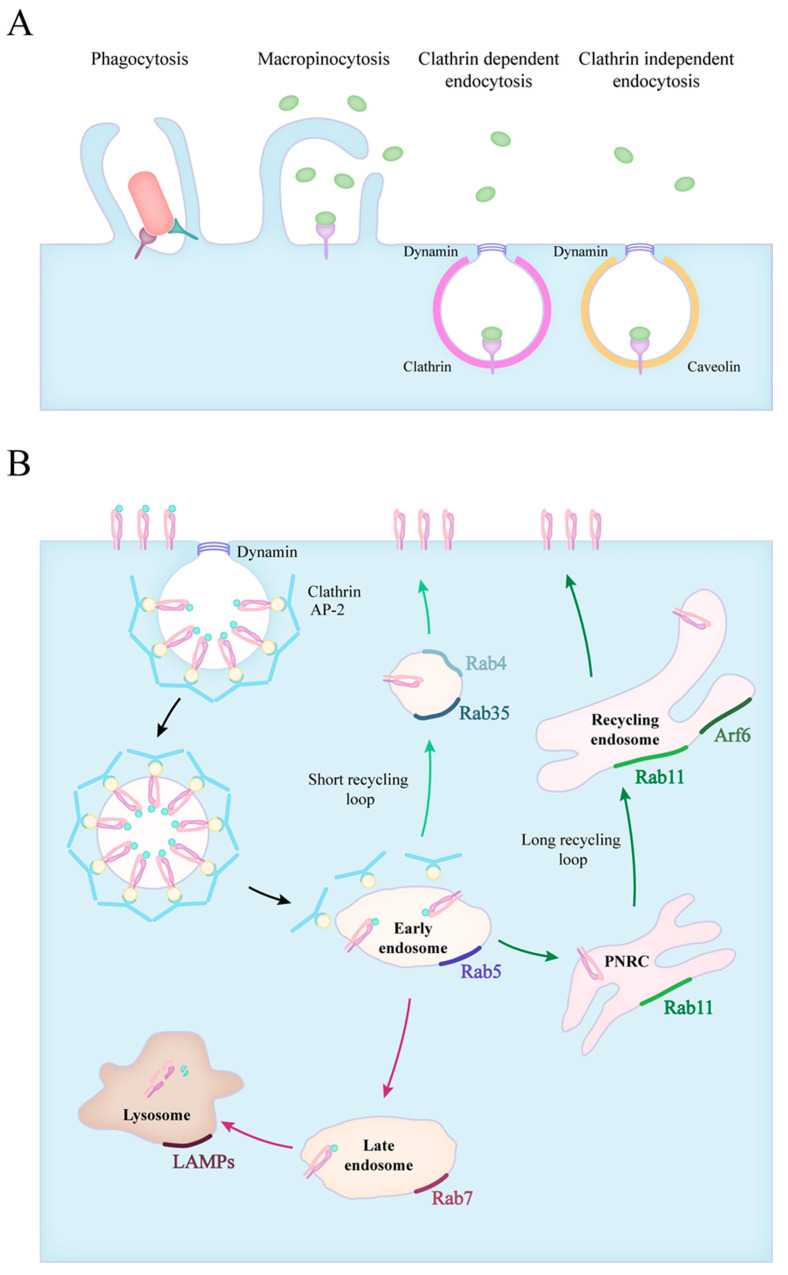
Forms of particle uptake and the endocytic routes. (**A**) The endocytic routes involve phagocytosis, macropinocytosis, clathrin mediated and clathrin independent endocytosis. (**B**) The fate of internalized particles is shown in the case of clathrin dependent endocytosis. Vesicles scissed from the plasma membrane quickly lose their clathrin coating and fuse with early endosomes (black arrows). Early endosomes transition into late endosomes, and in time, to lysosomes (red arrows). The recycling of receptors can occur via a short loop (green arrows), directly from early endosomes or through a long loop across the perinuclear recycling compartment (PNRC) and recycling endosomes (dark green arrows). The small GTPase Rab proteins characteristic for each stage are also indicated.

Various portals provide regulated entry into the cells, some of these mechanisms are constitutive, while others are based on receptor-mediated interactions. Macromolecules can enter the cell, enclosed into membrane-bound carriers, during the process of endocytosis. The known mechanisms of endocytosis differ in the mode of uptake and the intracellular pathway of the internalized cargo. These mechanisms include macropinocytosis, phagocytosis, clathrin-mediated and clathrin-independent endocytosis (Figure 1A) [19].

Cells can take up larger amounts of extracellular fluid and membrane-bound particles through the evolutionarily conserved macropinocytosis, with a vacuole size of 200–500 nm [20]. Macrophages and dendritic cells use this actin polymerisation driven method to constantly monitor their environment and collect peptides for antigen presentation [21].

Receptor-bound macromolecules and particles can be internalized through clathrin-mediated endocytosis (CME). The ligand–receptor complexes are concentrated into patches in the cell membrane, where clathrin scaffold proteins and various adaptor molecules are recruited to form clathrin-coated pits (CCP) on the cytoplasmic surface of the plasma membrane. Then the membrane closes and a small clathrin-coated vesicle (CCV) arises, forming a 60–120 nm vacuole [22]. The internalization of endosomes requires the rearrangement of the actin cytoskeleton and the GTPase dynamin performs the last step of the process. After the clathrin-coated pit sinks into the cytoplasm, dynamin creates a “collar” that closes the vacuole. The clathrin-coated vesicle is then detached from the plasma membrane and the disassembly of the endocytic machinery begins [23,24].

The many clathrin-independent pathways (CIE) are not as well characterized as CME, and they can be specified either by their characteristic cargo molecule or the coating of the vesicles. Lipid raft-associated molecules are often internalized in 50–100 nm large caveolin coated invaginations (caveolae), that are shuffled between the plasma membrane and early endosomes. Caveolae are most common in endothelial cells where they mediate transcellular transfer of serum proteins and nutrients from the bloodstream [25]. GPI-anchored proteins (GPI-APs) are internalized into the GPI-AP enriched early endosomal compartment (GEEC) in a dynamin- and coating protein-independent manner [26]. Other CIE pathways include the endocytosis of the IL-2 Receptor (IL-2R), the Arf6- and the Flotillin-associated routes [25,27].

Phagocytosis is defined as the specific receptor-induced uptake of particles larger than 500 nm, like pathogens, dead cells or foreign bodies. This process induces actin filament assembly that causes the formation of pseudopods that surround the antigen. The main steps of phagocytosis are: pathogens binding to receptors, receptor clustering, phagocytic cup formation and phagosome sealing. Actin filaments support the plasma membrane as it zips tightly around the particle forming the phagocytic cup. The plasma membrane and internal membranes—recycling endosomes, late endosomes, and endoplasmic reticulum (ER)—might contribute to the organization of the phagocytic cup, in a so-called focal exocytosis process [28].

## 3. Endocytic Route of Engulfed Particles and Receptor Recyclization

After antigen uptake, the newly formed phagosome undergoes fusion events with various cytosolic organelles resulting in phagosome maturation. During the process of phagocytosis the phagosomal contents are digested, recycled or exocytosed [29]. The self and non-self discrimination determine the steps of phagosome maturation [30]. The presence of pathogens induces the production of reactive oxygen and nitrogen intermediates in the lysosomes of activated phagocytes, where the terminal degradation of the internalized pathogen takes place [29]. Both self and non-self molecules are continuously sampled and presented to T cells, strengthening self-tolerance or inducing a protective immune response. T cells recognize the antigens in peptide–MHC-I and –MHC-II complexes. MHC-II molecules accumulate within the internal membranes of endosomes, where peptide loading occurs [31].

The maturation of phagosomes is brought forward by the fusion with vesicles from the endosomal system, which is controlled by the Rab family of small GTPases [32]. The members of this family show a characteristic distribution, therefore they are often used as markers for the different stages of endosome maturation (Figure 1B). Firstly, phagosomes acquire Rab5 which promotes their fusion with early endosomes through the recruitment of Early Endosome Antigen l (EEA1) [33,34]. The size of the early endosomes is balanced by the fissure of Rab4-positive recycling vesicles directed at the plasma membrane [35,36,37]. The membrane composition of vesicles dynamically changes during maturation, and Rab5 is quickly exchanged for Rab7, which now mediates fusion with late endosomes, and later, lysosomes [38,39]. At this stage, the pH inside the vacuoles starts to decrease with the help of the vacuolar-type proton pump ATPases (V-ATPases), and this is followed by the accumulation of luminal hydrolytic enzymes [40]. Ubiquitinated proteins destined for degradation are often sorted from early to late endosomes and lysosomes via tubular invaginations that form intraluminal vacuoles (ILVs) in multivesicular bodies (MVBs) [41]. Additionally, MVBs can also fuse with the plasma membrane that leads to the secretion of ILVs as exosomes, which are important elements of intercellular communication [42]. As the final step, the phagosome fuses with lysosomes, creating the phagolysosome, characterized by lysosome-associated membrane proteins (LAMPs) and a strongly acidic pH as low as 4.5. The acidic milieu of the phagolysosome activates the acidic protease cathepsins, which cleave the phagocytosed proteins into the smaller peptides necessary for antigen presentation on MHC complexes [43]. The recruitment of the NADPH oxidase complex also contributes to the elimination of ingested microbes through the production of reactive oxygen species [44].

The acidification, enzymatic activity and subsequent protein degradation are tightly regulated and often influenced by the activation and differentiation status of the antigen-presenting cells. For example, the IL-4/IL-13 induced maturation of macrophages (M2 or alternative phenotype) increases proteolytic activity while decreasing NADPH activity, developing a phagolysosome environment for quick protein degradation during tissue repair and wound healing. In contrast, LPS/IFNγ stimulated (M1) macrophages produce a higher amount of reactive oxygen species for the effective elimination of pathogens [45], and the lower acidity and proteolytic capacity helps to retain peptides for antigen presentation [46,47]. In the case of dendritic cells, Delamarre et al. showed that their lysosomes contained fewer proteolytic enzymes, resulting in a slower degradation and the retention of antigens in the lymph nodes, which is crucial for an effective antigen presentation [48,49]. These observations highlight a cell type-specific regulation of phagocytosis and the endocytic pathway, to better suit the distinctive functions of antigen-presenting cells.

The availability of receptors is regulated by continuous receptor trafficking, which can occur via distinct routes [50]. Instead of degradation, they are recycled back to the cell membrane, extending the lifetime of these proteins [51]. These recycling routes are most explored in the case of the transferrin receptor (TfnR) and integrins. The TfnR, α_V_β_3_- and inactive β_1_-integrins were shown to cycle through a short, Rab4-dependent pathway, where these receptors are quickly returned to the plasma membrane from the early endosomes [36,52,53]. In the Rab11-dependent long loop, described both for TfnR and β_1_-integrins, receptors go through the perinuclear recycling compartment (PNRC) before returning to the cell surface [54,55]. Other small GTPases, like Rab35 and Arf6 (ADP-ribosylation factor 6) were also implicated in the return of receptors to the cell surface [55,56,57]. The fast recycling of integrins and phagocytic receptors grants the possibility for continuous ECM and ligand engagement, indispensable for cell migration and pathogen elimination. 

Endocytosis is a highly conserved process among metazoans, thus most of the proteins involved in the endocytic trafficking in *Caenorhabditis* *elegans* possess the same name (ARF-6, RABs, etc.) and function as their human orthologs. Therefore, the nematode has been used early on as a model to monitor this process. Studies in *C. elegans* revealed various roles of membrane trafficking in development and physiology, and provided significant data at the molecular level of several mechanisms, including vesicle budding, membrane fission and fusion and cargo sorting [58]. As this organism is suitable for fast and efficient high throughput screens, plenty of studies identified new endocytosis-related genes, like *cdc-42* (Cell Division Cycle related) and *par-6* (abnormal embryonic PARtitioning of cytoplasm) [59]. Genetic analysis in coelomocytes, cells specialized for fluid uptake, also revealed a role for *cup-5* (Coelomocyte UPtake-defective, homologue of human mucolipin-1) in lysosomal function [60].

## 4. Phagocytic Receptors and Pathogen Recognition

### 4.1. Pattern Recognition and Opsonic Receptors in Mammals

Innate immune cells populate the possible entry points in the body and provide the first line of defence against invading pathogens. These cells utilize a range of receptors to quickly engage, capture and internalize their targets (Table 1). Pattern recognition receptors (PRRs) bind the pathogens directly via pathogen-associated molecular patterns (PAMPs), while complement or IgG opsonized targets are recognized by complement- and Fc-receptors, significantly increasing the efficiency of phagocytosis [61]. Additionally, these cells have receptors dedicated to sensing the signs of danger, the anaphylatoxic complement fragments C3a and C5a or chemokines produced by resident cells in inflamed tissues [62,63]. These receptors have an important role in initiating the immune response by promoting leukocyte recruitment, enhanced phagocytosis and the release of reactive oxygen species [64,65].

Pattern recognition receptors can be classified into several major families, but not all of them participate in particle internalization. Those that directly do not participate in phagocytosis modulate the immune response through signalling events. Toll-like receptors (TLRs) are transmembrane proteins found both intracellularly and on the cell surface, they recognise many bacterial, viral or fungal structures and danger signals from the host cells [66]. The intracellular NOD-like receptors (NLRs) recognise bacterial and fungal wall components and they have an important role in the induction of inflammation. Some NLRs are constituents of inflammasomes, where they trigger the activation of the pro-inflammatory cytokines IL-1β and IL-18 upon pathogen encounter [67]. The family of RIG-like receptors (RLRs) represents cytosolic receptors that bind viral nucleotides and elicit an inflammatory and antiviral response through the induction of type I IFN production [68]. C-type lectins (CLRs) are known to bind glycans through their carbohydrate recognition domain (CRD), mostly in a Ca^2+^ dependent manner, and with diverse structural and ligand binding features they recognise a wide range of pathogens [69]. Several CLRs were shown to be directly involved in the phagocytosis of microbes, while others might only help endocytosis through cell activation or ligand tethering.

The CLR family contains several phagocytic receptors: Dectin-1 provides anti-fungal immunity via the recognition and phagocytosis of β-glucan containing fungi—for example, *Aspergillus fumigatus*, *Candida albicans* or *Pneumocystis* [70,71], and it also participates in the binding of *Mycobacterium* and *Leishmania* species [72,73]. The signalling through dectin-1 induces cytokine and ROS production and regulates the release of NETs in neutrophils [74]. Dectin-2 recognises the mannose components of bacterial and fungal cell walls found in *C. albicans*, *Saccharomyces*, *Microsporum audouinii*, *Trichophyton rubrum* and *Mycobacterium tuberculosis* [75,76,77]. The priming of dectin-2 is indispensable for an effective anti-fungal response, as it was shown to promote cytokine production and Th17 differentiation after *C. albicans* ligation [78]. Mincle (Macrophage-inducible C-type lectin) binds glycolipids on pathogens like *Mycobacterium*, *Malassezia*, *Streptococcus pneumoniae* and *Klebsiella pneumoniae* or lipid-based damaged self molecules, helping in the maintenance of tissue homeostasis [79,80,81,82,83]. Mincle was found to be in a functional complex with another CLR, namely MCL (Macrophage C-type lectin), and they use FcεRIγ for signalisation. Mincle and MCL are both receptors for the mycobacterial cord factor TDM (trehalose-dimycolate) [84,85], and the association of these molecules enhances phagocytic capacity [86].

Many C-type lectins were described as a receptor for the Human Immunodeficiency Virus (HIV), which are used to infect cells or transmit the virus to interacting partners. The Dendritic Dell-Specific Intercellular adhesion molecule-3 Grabbing Non-integrin (DC-SIGN) binds highly mannosylated and fucoslyated glycoproteins, which are abundantly expressed by many pathogens, like *Mycobacterium tuberculosis*, *Helicobacter pylori*, *Leishmania mexicana*, and *Schistosoma mansoni* [87,88]. DC-SIGN also binds many other viruses, for example, Dengue virus, Ebola virus, SARS, Hepatitis C virus (HCV) and cytomegalovirus (CMV), which often utilize this connection to enter DCs [89,90,91,92,93]. The binding of HIV-1 to DC-SIGN enhances the trans-infection of CD4^+^ T cells that come in contact with the infected DCs migrating to the lymph nodes [94,95]. The next receptor worth mentioning is the DC Immunoreceptor (DCIR), also specific for fucose and mannose containing glycans, and with the ability to bind HIV-1, this receptor similarly promotes virus dissemination [96,97]. On macrophages, the receptor responsible for the transmission of HIV and Dengue virus was shown to be the Macrophage Mannose Receptor (MMR) [98,99]. MMR recognizes mannose, fucose, and GlcNAc residues, found in many fungal or bacterial walls and virus envelopes [100,101]. The phagocytic internalization mediated by MMR was demonstrated in the case of *Mycobacterium tuberculosis* and *Francisella tularensis* [102,103]. Langerin is a trimeric receptor expressed on Langerhans cells, and was shown to recognize several glycoprotein ligands, including mannosylated and sulfated glycans [104,105], fungal β-glucans in the wall of several *Candida* and *Saccharomyces* species [106] and viral envelope proteins of HIV-1 and measles virus [107,108]. The internalization and digestion of particles through langerin were proven in the case of a mannosylated glycoprotein [109] and HIV-1 particles [107]. In contrast to DC-SIGN, the langerin-mediated uptake of HIV-1 results in the degradation of the virus inside the Birbeck granules of Langerhans cells, preventing its transmission to T cells [107].

The many members of the scavenger receptor family contribute to the elimination of non-self and altered self molecules by various functions and a wide spectrum of ligands. Some of them were also indicated in the recognition and engulfment of apoptotic cells [110]. Based on the latest attempt at creating a consensus nomenclature for scavenger receptors, 12 classes (A–L) were distinguished based on structural features, a full list and characterization of these molecules were reviewed by PrabhuDas et al. [111].

The first discovered Class A receptors entail the Macrophage Scavenger Receptor (MSR1, SR-A1) and the Macrophage Receptor with Collagenous Structure (MARCO, SR-A6). They are characterized by an α-helical coiled-coil, a collagenous, and a C-terminal scavenger receptor cysteine-rich (SRCR) domain [112]. Their ligands include the modified or oxidized, but not native LDL, and Gram-positive and -negative bacterial wall constituents like lipoteichoic acids and lipid A [113,114,115,116]. MARCO is predominantly expressed on macrophages in the lungs and in the spleen where its main function is the clearance of pathogens [117,118].

One of the most studied scavenger receptors is CD36, representing Class B molecules, with a structure of two transmembrane domains connected by an extracellular loop [119]. It recognizes oxidized LDL, β-amyloids and polyanionic ligands of many bacteria [120,121,122] and cooperates with α_v_β_3_ and α_v_β_5_ in the elimination of apoptotic cells [123,124].

The F Class includes SCARF-1 (Scavenger Receptor Class F Member 1, SR-F1), and MEGF10 (Multiple EGF Like Domains 10, SR-F2), with an extracellular region built of EGF–like repeats [125]. SCARF-1 binds bacterial and fungal pathogens and heat-shock proteins [126,127] and similarly to its *C. elegans* ortholog CED-1, it is involved in the engulfment of apoptotic cells [128]. MEGF10 binds both β-amyloids and C1q on apoptotic cells in the brain, indicating a possible role in the pathogenesis of neurological disorders [129,130].

The Class H Stabilin-1 and Stabilin-2 consist of fasciclin, EGF-like, and lamin-type EGF-like domains (FEEL). Both receptors were indicated in the PtdSer mediated phagocytosis of apoptotic cells [131,132]. Additionally, Stabilin-1 was also shown to bind Gram-positive and -negative bacterial ligands [133], whereas Stabilin-2 is important in the turnover of hyaluronic acid and chondroitin sulphate, and it interacts with α_v_β_5_ to promote the engulfment of damaged erythrocytes [134,135].

The second branch of pathogen clearance is opsonophagocytosis. This process utilizes the immediate deposition of complement fragments on microbial surfaces, and later on, the binding of specific antibodies. Antigens opsonised with complement fragments and immunoglobulins become available for uptake via complement- and Fc-receptors, enhancing phagocytic capacity, or in some cases, it can provide the pathogens the opportunity to infect host cells [136]. The activation of the complement cascade results in the cleavage of C3 and C4 molecules, which generates the ligands for complement receptors: C3b and C4b for CR1, iC3b for CR3 and CR4, and CRIg binds both C3b and iC3b. The binding site of CRIg on iC3b and C3b does not overlap with the residues recognized by CR1, CR3 or CR4, thus providing the possibility for cooperation between these receptors [137].

CR1 is a single chain transmembrane glycoprotein composed of Short Consensus Repeat domains (SCRs). This receptor is involved in the clearance of complement opsonized pathogens and immune complexes (ICs) from blood. Red blood cells bind complement-containing ICs via CR1, then deliver them to the liver or spleen for elimination [138]. CR1 was shown to be a receptor for Epstein–Barr virus (EBV), opsonized HIV and bacteria, often in cooperation with CR3 [139,140,141].

CRIg is expressed by resident macrophage subsets, among which the most studied are Kupffer cells in the liver. It is proposed that Kupffer cells quickly clear circulating pathogens from the blood in an anti-inflammatory manner, without the involvement of other PRRs and CRs, until pathogen numbers rise above a limit [137]. The importance of CRIg in the rapid internalization of pathogens was proven in the case of complement opsonized Adenovirus particles, *Staphylococcus aureus*, *Listeria monocytogenes* and *Candida albicans* [142,143,144].

The β_2_-integrins CR3 and CR4 are known to play an essential role in cell motility and the elimination of pathogens, tumour- and apoptotic cells via phagocytosis [145]. The many ligands described for these receptors, including complement proteins, ECM components and adhesion molecules, are all recognised by the major ligand-binding site in the αI-domain [146]. The β-glucan components of fungal cells are recognized by a separate lectin site in CR3, located C-terminally from the I-domain [147,148]. An also I-domain independent binding site for bacterial lipopolysaccharides was shown as well, in both CR3 and CR4 [149,150]. Due to their structural similarities, the two receptors are often assumed to have the same functions, but depending on the cell type and microbe used in the experiments, there can be differences in their pathogen recognition. Both receptors were implicated in the uptake and killing of *Escherichia coli* and *Mycobacterium tuberculosis*, but in the case of *Staphylococcus aureus* only CR3 participated in the phagocytosis [151,152,153,154]. The list of pathogens interacting with complement receptors has been previously reviewed by our group [136].

### 4.2. Innate Immunity and the Lack of Direct Pathogen Recognition in C. elegans

Innate immunity is the evolutionarily ancient part of the immune system that is already present in invertebrates. It is important to note however that in *C. elegans*, the engulfment and elimination of dying cells are not mediated by professional phagocytes, but instead, they are performed by neighbouring cells, which can be hypodermal, muscle, intestinal and gonadal sheath cells [165,166].

In the nematode, the innate immune system is activated through the recognition of cell damage via damage-associated molecular patterns (DAMPs) that can be both pathogen-induced or environmental in origin. Abiotic stress factors, for example, altered temperature or changes in osmolarity, provoke an unfolded protein response or result in the accumulation of reactive oxygen species. Recent data show that there is an interplay between signalling pathways mediating stress responses and the activation of innate immunity, as perturbations of basic cellular mechanisms, such as translation or mitochondrial function also result in cellular damage [3]. Thus, the above process, which is also known as surveillance immunity [167], only allows the worm to fight against microbes after the infection has already taken place and the damage has to be cured.

Following the recognition of DAMPs, downstream signal transduction pathways are activated, which leads to changes in the expression of genes encoding antimicrobial peptides (AMPs) and other secreted signals mediating the effector mechanisms of the innate immune response. Specifically, infection recognition activates evolutionarily conserved signal transduction ways, including the mitogen-activated protein kinase (MAPK) signalling, the DAF-2 (Abnormal Dauer Formation-2)/insulin-like receptor or the DPP/BMP Like-1 (DBL-1)/transforming growth factor β (TGF-β) pathways [3]. The stress-activated MAPKs, JNK (c-Jun *N*-terminal kinase) and p38, play a crucial role in the regulation of both innate immune defence and abiotic stress responses in mammals [168,169]. The *C. elegans* p38 MAPK homologue PMK-1 (p38 MAP Kinase Family-1) and its upstream activating kinases SEK-1 (SAP/ERK kinase-1 as MAPKK/MAPK kinase) and NSY-1 (Neuronal Symmetry-1 as MAPKKK/MAPK kinase kinase) were first shown to function in host resistance to *Pseudomonas aeruginosa* in the intestine [170,171]. Later studies revealed the central role of this MAPK cascade in defence mechanisms against a broad range of other bacterial and fungal pathogens in the intestine and epidermis [172,173,174,175,176]. KGB-1 (Kinase GLH Binding-1), the worm JNK-like MAPK homologue, is involved in the protection from bacterial pore-forming toxins [177]. The third MAPK module is also implicated in host defence mechanisms in the worm. Together with the Ras homologue LET-60 (LEThal-60), LIN-45 (abnormal cell LINeage-45, homologous to Raf), MEK-2 (MAP kinase kinase-2) and MPK-1 (MAP Kinase-1) are required to induce the tail-swelling response against *Microbacterium nematophilum* in rectal epithelial cells [178].

Despite similarities in downstream signalling cascades, initial pathogen recognition does not function in a conserved manner between *C. elegans* and mammals. Many pattern recognition receptors found in other species are missing in the worm [179] (for example, NLRs or peptidoglycan recognizing receptors), and although the worm genome encodes a large set of galectins and C-type lectins [180], their role in pathogen recognition has not been confirmed. The nematode displays a sole Toll-like receptor homologue TOL-1, but this molecule does not function directly in the recognition of pathogens [181,182]. Instead, it is involved in pathogen-avoidance behaviour by promoting the development and function of chemosensory BAG neurons, which monitor the metabolic activity of microbes [183]. In contrast, TIR-1 (Toll and Interleukin-1 Receptor domain protein-1), orthologous to the human TIR domain-containing SARM (sterile α and armadillo motif-containing protein) [181,184], is directly involved in innate immune response as it is required for PMK-1 activation upstream of NSY-1 in host resistance against many pathogens [185].

According to Matzinger’s danger theory, the innate immune system is activated not only by pathogen-associated molecular patterns (PAMPs) but also by tissue-derived danger signals, called damage-associated molecular patterns (DAMPs). This is relevant because *C. elegans* lacks PRRs, implying that the innate immune response is also triggered by cellular damage [186,187]. Indeed, Pujol et al. showed that injuries of the epidermis caused by a needle result in PMK-1 activation reminiscent of that observed after a fungal infection [188]. A similar effect was detected in the intestine where the direct cellular damage caused by bacterial pore-forming toxins also activated PMK-1 [177,189].

A recent study conducted by Zugasti et al., investigating the regulation and induction of antimicrobial peptides (AMPs) in the epidermis, demonstrated how pathogen infection caused by *Drechmeria coniospora* and direct wounding of the epidermis are connected [190]. The pathogen’s conidia adhere to the worm’s cuticle, penetrate it, disrupting epidermal integrity. They found that a G protein-coupled receptor (GPCR), DCAR-1 (DihydroCaffeic Acid Receptor-1) is activated in response to physical injury of the epidermis caused by *Drechmeria* infection. Downstream of DCAR-1 the signal is mediated by Gα and Gβ encoding genes *gpa-12* (G protein alpha subunit-12) and *rack-1* (RACK-1 (mammalian Receptor of Activated C Kinase) homolog) [191], which activate *tpa-1 (tetradecanoyl phorbol acetate resistant-1)*, homologous to the mammalian protein kinase C δ (PKCδ). Subsequently, TPA-1 acts through the p38 MAPK pathway (TIR-1/NSY-1/SEK-1/PMK-1) and involves the STAT-like transcription factor STA-2 (STAT Transcription Family 2) [192] to regulate a cluster of *nlp* (neuropeptide-like protein) genes such as *nlp-29*, encoding antimicrobial peptides [181,193]. Zugasti et al. identified an endogenous ligand of DCAR-1, hydroxyphenyllactic acid (HPLA), a tyrosine derivative whose level is elevated upon fungal infection and direct wounding of the epidermis. It remains to be solved whether the pathogen *D. coniospora* directly increases HPLA levels or the elevated level of the tyrosine derivative is the response of the worm for the physical damage in the epidermis upon infection. Both scenarios support the idea that HPLA acts as a DAMP for the nematode [190].

## 5. Phagocytosis of Apoptotic Cells

### 5.1. Apoptotic Engulfment in Mammals

The efficient clearance of senescent and dying cells during ontogenesis and aging is crucial for the maintenance of tissue homeostasis. The phagocytosis of apoptotic cells, termed efferocytosis, is considered to be immunologically silent or even immune-suppressive, with tissue-resident macrophages releasing anti-inflammatory mediators and dendritic cells inducing the differentiation of tolerogenic T cells after the uptake of apoptotic cells [4,194,195].

Although cells undergoing apoptosis retain an intact plasma membrane, there are changes in the phospholipid composition in the outer leaflet that work as an “eat me” signal for phagocytes. The most well-known indicator of apoptosis is the exposure of phosphatidylserine (PtdSer) on the cell surface [196]. In healthy cells, this phospholipid is actively kept in the inner leaflet by lipid transporters called flippases [197]. The caspases present during apoptosis both disrupt the function of flippases maintaining the asymmetrical distribution of PtdSer and activate scramblase enzymes that catalyse the reverse translocation of this molecule [198,199].

**Figure 2:**
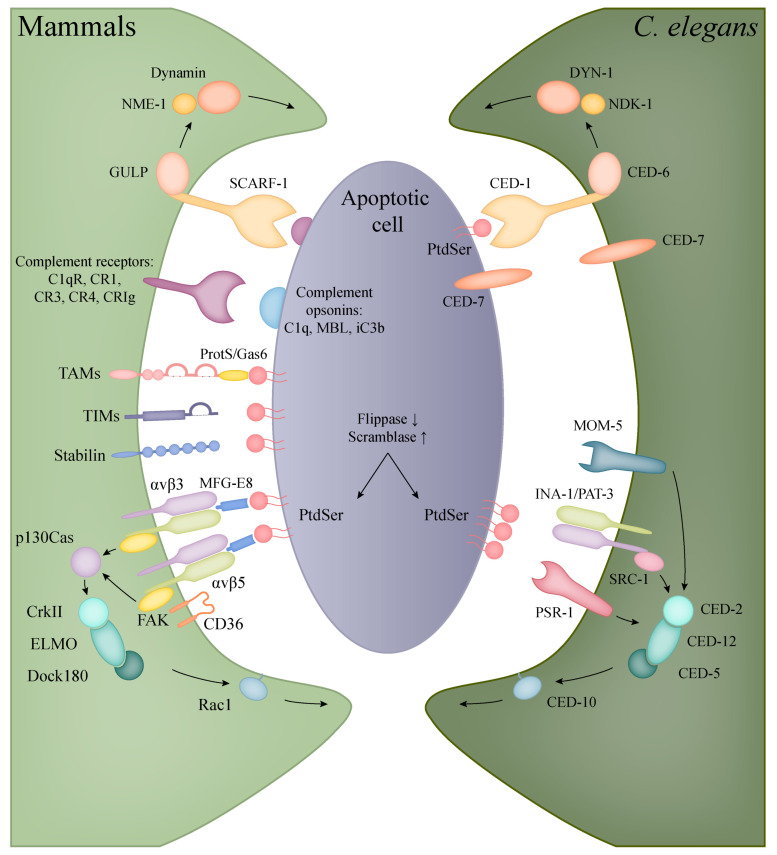
Recognition and engulfment of apoptotic cells in mammalian phagocytes and *C. elegans*. The evolutionarily conserved CED-1/CED-6/DYN-1 (corresponding to human SCARF-1/GULP/Dynamin) and CED-2/CED-5/CED-12/CED-10 (in human: CrkII/Dock180/ELMO/Rac1) pathways are present in both *C. elegans* and mammals with the same functions in the clearance of apoptotic cells (orthologous molecules are indicated with the same colours). In mammals, however, a higher diversity can be observed in the recognition of apoptotic bodies, including the families of TIM and TAM proteins, complement and scavenger receptors.

PtdSer is either directly recognised or is bound to receptors on phagocytes through soluble bridging molecules (Figure 2, Table 2). A PtdSer receptor (PSR) was first described by Fadok et al. using a monoclonal antibody (mAb217) that bound to the surface of macrophages and inhibited the uptake of apoptotic bodies [200]. However, subsequent experiments trying to identify this receptor produced some contradictory data [201]. The protein encoded by the *psr* gene, later named JMJD6 (Jumonji domain-containing protein 6), was shown to be located in the nucleus, having an important function in embryonic development with a histone demethylase activity [202,203]. Thus, it is possible that the surface molecule responsible for the binding of apoptotic bodies is not identical to the product of the suspected evolutionarily conserved *psr-1* gene.

The receptors of the TIM family (T cell immunoglobulin- and mucin-domain-containing molecule) mediate the recognition and/or uptake of apoptotic bodies in various cell types by directly binding PtdSer. All three human TIM proteins (TIM1, -3, -4) have been proven to bind PtdSer, but the internalization of particles can be cell type-specific. TIM1 functions on T_h_2-cells as a costimulatory molecule [204] and it is upregulated in kidney epithelial cells after injury, allowing the engulfment of apoptotic bodies for tissue recovery [205]. TIM3 is expressed on T_h_1 cells and dendritic cells in humans, but the internalization of PtdSer containing apoptotic bodies was only observed in myeloid cells [206,207,208]. Presumably, the most efficient apoptotic cell receptor of this family is TIM4 that is expressed on dendritic cells and various tissue-resident macrophages, like peritoneal or liver Kupffer cells, providing clearance throughout the body [209,210,211].

The indirect binding of PtdSer can happen through the bridging molecules Protein S and GAS6 (growth-arrest-specific 6) and their receptors, the tyrosine kinase TAMs (Tyro3, Axl, Mer) [212]. Protein S is synthetised mostly in liver hepatocytes and activates Tyro3 and Mer, but not Axl, whereas GAS6 acts as an agonist for all three TAM receptors [213]. The TAM receptors are expressed in both macrophages and dendritic cells, but Seitz et al. showed that DCs mainly rely on the functions of Axl and Tyro3, whereas macrophages use all three [214]. The signalling through TAMs contributes to tissue homeostasis via the inhibition of inflammatory responses in myeloid cells [215,216].

Another group of soluble PtdSer-recognizing molecules includes MFG-E8 (milk fat globule-EGF factor 8 or lactadherin) and thrombospondin, which contain RGD motifs, thus connecting apoptotic cells to the integrins α_v_β_3_ and α_v_β_5_ [217,218]_._ Both integrins were shown to cooperate with the scavenger receptor CD36 in the uptake of apoptotic cells [124,219]. The binding of apoptotic cells through these integrins initiates the phosphorylation of FAK and the recruitment of p130^Cas^ (Crk-associated SRC substrate), which connects to the evolutionary conserved CrkII–Dock180-ELMO-Rac1 pathway mediating the engulfment of the particles through actin polymerisation and phagocytic cup formation [220,221,222]. Other scavenger receptors are also known to participate in the elimination of apoptotic cells, the specific ligands and functions of this receptor family are detailed in Section 4.1.

The complement system also participates in the opsonization, recognition and elimination of apoptotic cells. The complement components C1q and MBL bind apoptotic and necrotic cells, enhancing their uptake by phagocytes and in the presence of serum, activating the classical and lectin pathway [223,224,225]. The activation of the alternative pathway by apoptotic cells was also proved by several studies, resulting in the deposition of C3 fragments and phagocytosis by the complement receptors CR1, CR3, CR4 and CRIg [226,227,228]. Opsonization with complement facilitates the quick and effective clearance of apoptotic cells without an inflammatory response, which is further supported by the immunosuppressive qualities of C1q [229,230].

### 5.2. Apoptotic Engulfment and Clearance in C. elegans

Programmed cell death can be divided into three phases: the first is the specification of the cell destined to die, and the second is the killing phase, where the apoptotic pathway is activated and lastly the execution of cell death and dismantling. The last phase also includes the elimination of apoptotic bodies, where a specialized or neighbouring cell engulfs and removes the debris of the dying cell. The conserved genes involved in the core apoptotic pathway were first identified in *C. elegans*, for which the Nobel prize for Medicine was awarded to Sydney Brenner, John E. Sulston, and H. Robert Horvitz in 2002 [15,16]. The main characteristics and genetic pathways of apoptosis in *C. elegans* are detailed in this special issue published by E. Pourkarimi.

Phagocytosis of apoptotic corpses involves conserved pathways in the nematode (Table 2). The worm does not possess dedicated macrophages; instead, the neighbouring cells provide the phagocytic function. Similar to other organisms, the well-conserved PtdSer acts as the main signal of cell death or “eat-me” signal. The exposure of PtdSer in the worm during apoptosis is linked to the amino phospholipid translocase TAT-1 (Transbilayer Amphipath Transporter, ortholog of human flippase, ATPase phospholipid transporting 8A2, ATP8A2) and CED-8 (CEll Death abnormality 8, ortholog of human scramblase XKR8) function. In a living cell translocases actively transport PtdSer from the outer to the inner leaflet of the plasma membrane, but in an apoptotic cell, the inactivation of translocases and the activation of scramblases results in PtdSer accumulation on the cell surface [231]. After the recognition of an apoptotic cell, the activated receptors stimulate the extension of the engulfing cell’s membrane and the rearrangement of the actin cytoskeleton. Consequently, the phagocytic cell develops pseudopods around the dying cell. Just as the pseudopods fuse, the newly formed phagosome separates from the plasma membrane [232].

Two main, partly overlapping and conserved signalling pathways control the engulfment of apoptotic cells in *C. elegans* [233]. One contains the phagocytic receptor CED-1 (human SCARF-1), the adaptor CED-6 (GULP), the ABC transporter CED-7 (ABCA) and the large GTPase DYN-1 (Dynamin2). The second path entails the CED-2 (CrkII), CED-5 (Dock180), CED-10 (Rac1), CED-12 (ELMO) proteins, the latter being the counterpart of the human Rac signalling. These two pathways partly converge at CED-10 involved in actin polymerisation, regulating the required cytoskeleton rearrangement for engulfment [234,235].

The first pathway is triggered by the phagocytic transmembrane receptor CED-1, which only appears on the surface of the engulfing cell. After ligand recognition, the amount of CED-1 increases in the engulfing cell’s plasma membrane that is in contact with the neighbouring dead cell, initiating the formation of the phagocytic cup. The PtdSer driven activation of CED-1 triggers the subsequent members of the signalling cascade, resulting in the growth of pseudopods [236]. CED-7 has a dual role to mediate the eat-me signal, it is expressed in both the engulfed and the engulfing cell: CED-7 assists in the exposure of PtdSer and also helps CED-1 to capture the PtdSer signal [237]. Then the activated CED-1 signal is transmitted by CED-6, partly activating CED-10, but mostly triggers through DYN-1 that regulates the extension of the engulfing cell membrane [234].

DYN-1 is a large GTPase that has multiple roles related to membrane trafficking. During engulfment, the phagocytic cup is transiently enriched in DYN-1 that provides the surplus of membrane necessary for pseudopod extension by helping the recruitment of early endosomes and their fusion with the cell membrane [238]. Furthermore, it also mediates the last event in the engulfment process, namely the fission of phagosomes from the plasma membrane [23]. For these functions, DYN-1 requires high amounts of GTP, which is provided by NDK-1 (Nucleoside Diphosphate Kinase-1, ortholog of human NME1) [239]. It is important to note that both DYN-1 and NDK-1 are detected on the surface of early phagosomes, indicating that they also have roles in the early steps of phagosome maturation [238,239,240].

The second pathway has an equally important role in the engulfing process: next to the membrane extension detailed above, the rearrangement of actin cytoskeleton is also crucial for the internalization of apoptotic cells. The components of this pathway regulate CED-10 activation [241]. The pathway is triggered by the recognition of PtdSer by three different receptors: integrins, PSR-1 and MOM-5. The core of this pathway is the CED-2/CED-5/CED-12 trio, which complex acts as the guanine nucleotide exchange factor (GEF) of CED-10. The first possible route to trigger this pathway is through the integrin consisting of two subunits: INA-1 (integrin alpha-1) and PAT-3 (Paralysed Arrest at Two-fold, beta subunit) [242,243]. In this case, CED-2 connects to the integrin receptor through SRC-1 (SRC oncogene related) and recruits the other two members of its complex [242]. The other alpha subunit, PAT-2, might also have a role in recognising PtdSer, but the results published about the exact role of the PAT-2/PAT-3 heterodimer receptor in phagocytosis is controversial [244,245]. The next receptor that has an influence on this pathway is PSR-1 (ortholog of human PSR, phosphatidyl serine receptor). CED-12 directly connects to this receptor and recruits the other two members of its complex upon activation [243]. Besides the above-mentioned two receptors, the Frizzled homolog MOM-5 (More Of MS 5) connects the Wnt signalling to the engulfment process, which also acts through the CED-2/CED-5/CED-12 complex [246].

Recently, a third pathway has been identified, revealing the important role of RAB-35 in the early steps of apoptotic cell phagocytosis. RAB-35 is a multifunctional GTPase that plays important roles in phagocytosis, cytokinesis, apico-basal polarity, cell migration, neurite outgrowth, immune synapse, exosome release and pathogen hijacking [247]. During phagocytosis, RAB-35 localizes at the developing pseudopods, and later on early phagosomes, suggesting a function in early phagocytic events [248]. The Zhou laboratory identified a novel role for RAB-35: *rab-35* mutants show a delay in apoptotic cell recognition, also *rab-35* mutant phenotypes are enhanced in both *ced-1* and *ced-5* mutant backgrounds, which is further worsened in *ced-1*, rab-35, ced-5 triple mutants. As a conclusion, *rab-35* functions in a third pathway in parallel to the *ced-2/-5*/*-10*/*-12* and *ced-1*/*-6*/*-7* pathways, and further genetic epistasis analysis indicates that *rab-35 function is also linked to* the integrin pathway [248].

### 5.3. The Interconnection of Innate Immunity with Apoptotic Cell Clearance and the Nervous System

Phagocytes perform a dual role in host defence as they are both responsible for removing apoptotic debris and initiating an innate immune response against pathogens. Despite its importance, the connection between these two functions is poorly understood. Recently it was shown in *C. elegans* that mutations in the genes involved in apoptotic cell clearance make them more resistant to the pathogenic bacteria *Pseudomonas aeruginosa* and *Salmonella typhimurium* [18]. The mutant worms showed an upregulation of the innate immune response genes, even in the absence of bacterial pathogens, and this response was actively regulated by the apoptotic cell clearance defects through the PMK-1 and MPK-1 pathways [18].

Similar observations were documented in mammals: inefficient removal of dead cells activates the innate immune system [249]. In DNaseII knockout mice, the DNA of apoptotic cells is not entirely degraded in lysosomes, which leads to the induction of an immune response and the development of chronic arthritis and anaemia [249]. In line with these data, the production of antibacterial peptides was observed both in *C. elegans* and *Drosophila* if apoptotic germ cells lacked DNaseII [250,251]. As there are some controversial data [252], further investigation is needed to precisely determine the relationship between the innate immune response and apoptotic cell clearance.

Interestingly, recent studies also suggest a neurological connection to immunity, as worms were proven to be able to avoid pathogen attacks through sensing microbes via their nervous system. Multiple sensory neurons and GPCRs are involved in the detection of specific molecules and the local fluctuations of oxygen and carbon dioxide levels generated by bacterial metabolism (reviewed in [253]). Thus, it is thought that the nervous system can regulate immunity in the nematode, in line with similar findings observed earlier in mammals [254,255,256]. *C. elegans* has only 302 neurons with detailed functional and morphological characterization available, and together with the knowledge of the entire synaptic wiring diagram, it can provide useful insights into the research of vertebrate neuroimmunology.

## 6. Discussion

In mammals, phagocytosis plays a dual role in immune defence. Firstly, pathogens are recognized and internalized by professional phagocytes, initiating the first steps in the fight against infection and later on, antigen presentation that promotes the induction of an adaptive immune response. Secondly, phagocytosis also provides the means to eliminate dead cells or cell debris to support tissue remodelling and to avoid autoimmune reactions.

Phagocytosis is an evolutionarily ancient defence mechanism that is already present in invertebrates. In this review, we compared elements of phagocytosis in mammals and in the nematode *Caenorhabditis elegans*. Although the two species utilise different mechanisms in the initial recognition of pathogens, the process of apoptotic cell engulfment is highly conserved between worms and mammals, making *C. elegans* a well-recognized model organism to study cell death [16]. Not only are the genetic cascades of the core apoptotic machinery conserved between humans and worms, but many regulators of cell clearance have homologs in mammals [241]. As *C. elegans* is often used for large-scale drug screens in many biological aspects [257], conserved components of the phagocytic machinery potentially allow using the worm as a model to test drugs, which enhance or inhibit phagocytosis.

Although *C. elegans* lacks the diverse machinery and immunological memory of vertebrates, it has an innate immune defence system and responds to infection by activating the expression of antimicrobial peptides [3]. However, the recognition of pathogens differs in the worm compared to mammalian systems. In the absence of PRRs, DAMPs are recognized instead of PAMPs, and consequently, the innate immunity is activated by cellular damage, which can be the result of many abiotic stress factors as well.

Ever-growing evidence proves that the immune system does not function autonomously, but it is interconnected with many cellular processes to maintain homeostasis. For instance, the field of neuroimmunology has been dynamically developing during recent decades after the initial discoveries that connect the two systems, such as the presence of neuroendocrine hormone receptors on immune cells, the innervation of lymphoid organs or Tracey’s inflammatory reflex theory (reviewed in [258]). However, studying the precise mechanisms of how individual neurons and neural circuits exert their effect on the immune system is challenging due to the complexity of the human nervous system, which is why *C. elegans* has been applied as a simple and useful model. With the well-characterized and defined number of neurons, this organism will enable the dissection of neural-immune signalling at a single cell level in the future [253].

## Figures and Tables

**Table 1:** Phagocytic receptors in mammals.

Receptors	Recognized Molecules	Distribution
**Pattern recognition receptors**		
CD14	LPS [155]	Mo, Mφ, DC, Neu
*C-type lectins*		
langerin (CD207)	sulfated and mannosylated glycans [104,105], fungal β-glucans [106], viral envelope glycoproteins [107,108]	LC
DC-SIGN (CD209)	fucosylated-, oligomannose- and *N*-glycans [87], HIV-1 [94]	DC
Dectin-1 (CD369)	fungal β-glucans [70,71], *Leishmania* [72], *Mycobacterium*	Mo, Mφ, DC, B cell, PMN
Dectin-2	mannose in fungal and bacterial cell walls [75,76,77]	Mo, Mφ, DC, Neu, B cell
MMR (CD206)	mannose, fucose, GlcNAc [100,101]	Mφ, DC
Mincle-MCL	fungal and bacterial glycolipids [81,82]	Mo, Mφ, DC, B cell
MGL	terminal GalNAc residues [156]	DC
DCIR	fucose and mannose containing glycans, HIV-1 [96,97]	Mo, Mφ, DC, PMN
*Scavenger receptors*		
MSR1 (SR-A1)	modified LDL, β-amyloid, heat-shock proteins [113,114], bacterial wall components [115,116]	Mo, Mφ, DC, MC
MARCO (SR-A6)	LPS, acetylated LDL [157,158]	Mφ
CD36 (SR-B2)	oxidized LDL, β-amyloids [120], polyanionic bacterial ligands [122], apoptotic cells [123]	Mo, Mφ, platelets
SCARF-1 (SR-F1)	bacterial, fungal pathogens, heat-shock proteins, apoptotic cells [126,127,128]	Mφ, DC, endothelial cells
MEGF10 (SR-F2)	C1q on apoptotic cells, β-amyloids [129,130]	astrocytes
Stabilin-1 (SR-H1)	PtdSer [131], bacterial components [133]	macrophages, endothelial cells
Stabilin-2 (SR-H2)	PtdSer [132], hyaluronic acid [134], heparin [159]	sinusoidal endothelial cells.
**Complement receptors**		
CR1 (CD35)	C3b, C4b [160], EBV [139], MBL, C1q [161]	Mo, Mφ, DC, PMN, NK, B cell, T cell subsets, RBC, FDC
CRIg	C3b, iC3b [143]	KC and tissue-resident macrophage subsets
CR3 (α_M_β_2_, CD11b/CD18)	iC3b [146], FH, FHR-1 [162], β-glucan [148], LPS [149]	Mo, Mφ, DC, PMN, NK, lymphocyte subsets
CR4 (α_X_β_2_, CD11c/CD18)	iC3b [163], FH [164], LPS [150]	Mo, Mφ, DC, PMN, NK, lymphocyte subsets

Abbreviations: DC: dendritic cell; FDC: follicular dendritic cell; FH: factor H; FHR: factor H related protein; KC: Kupffer cell; LC: Langerhans cell; LDL: low-density lipoprotein; LPS: lipopolysaccharide; MC: mast cell; MFG-E8: milk fat globule-epidermal growth factor 8; Mo: monocyte; Mφ: macrophage; Neu: neutrophil granulocyte; PMN: polymorphonuclear cells; PtdSer: phosphatidylserine; RBC: red blood cell.

**Table 2:** Molecules involved in the phagocytosis of apoptotic cells in human and *C. elegans*.

Human Protein	Function	*C. elegans* Protein
ATP8A2	P4-type ATPase/flippase	TAT-1
XKR8	scramblase	CED-8
SCARF1, MEGF10 and 11, LRP1 (CD91), Jedi-1	phagocytic receptor	CED-1
GULP	adaptor	CED-6
NME1	nucleoside diphosphate kinase	NDK-1
Dynamin	large GTPase	DYN-1
ABCA1 and ABCA7	ABC transporter	CED-7
JMJD6 (PSR?)	receptor	PSR-1
FZD1 and 7 (Frizzled class receptor 1 and 7)	receptor	MOM-5
integrin α/β chain	receptor	INA-1/PAT-3
SRC	non-receptor tyrosine kinase	SRC-1
CrkII	adaptor	CED-2
ELMO	adaptor	CED-12
Dock180	Rac GEF	CED-5
Rac1	Rho family GTPase	CED-10
MFG-E8	bridging between PtdSer and integrins	-
TIM1, 3, 4	PtdSer receptor	-
Protein S, GAS6	bridging between PtdSer and TAM receptors (MER, AXL, TYRO3)	-
